# Cucurbitacin covalent bonding to cysteine thiols: the filamentous-actin severing protein Cofilin1 as an exemplary target

**DOI:** 10.1186/1478-811X-11-58

**Published:** 2013-08-14

**Authors:** Mads Gabrielsen, Maike Schuldt, June Munro, Dagmara Borucka, Jenifer Cameron, Mark Baugh, Andrzej Mleczak, Sergio Lilla, Nicholas Morrice, Michael F Olson

**Affiliations:** 1 Beatson Institute for Cancer Research, Garscube Estate, Switchback Road Glasgow G61 1BD, UK; 2 Max Planck Institute of Molecular Cell Biology and Genetics, Pfotenhauerstrasse 108, 01307 Dresden, Germany

**Keywords:** Actin, Cofilin, Cytoskeleton, Cucurbitacin

## Abstract

**Background:**

Cucurbitacins are a class of triterpenoid natural compounds with potent bioactivities that led to their use as traditional remedies, and which continue to attract considerable attention as chemical biology tools and potential therapeutics. One obvious target is the actin-cytoskeleton; treatment with cucurbitacins results in cytoskeletal rearrangements that impact upon motility and cell morphology.

**Findings:**

Cucurbitacin reacted with protein cysteine thiols as well as dithiothreitol, and we propose that the cucurbitacin mechanism of action is through broad protein thiol modifications that could result in inhibition of numerous protein targets. An example of such a target protein is Cofilin1, whose filamentous actin severing activity is inhibited by cucurbitacin conjugation.

**Conclusions:**

The implications of these results are that cucurbitacins are unlikely to be improved for selectivity by medicinal chemistry and that their use as chemical biology probes to analyse the role of specific signalling pathways should be undertaken with caution.

## Background

The natural compound cucurbitacin triterpenes are a group of structurally related compounds that give a variety of plants and fungi their bitter taste as a defense against being eaten [1]. Historically, cucurbitacin-producing plants or extracts have been used as traditional remedies for diseases such as cancer, inflammation and infection [2]. More recently, cucurbitacins have attracted attention because of several notable properties. Their potent cytotoxicity has led to numerous investigations on their potential utility as anti-cancer therapeutics [3-5]. In addition, they have been used as chemical biology probes to explore the biological roles of signalling pathways including Jak/STAT3 [6,7], NF-κB [8], MAPK/ERK [9-11] and PI3-kinase [10]. Cucurbitacin analogues also have marked effects on the actin cytoskeleton, which in turn affects processes such as cell motility, tumour cell invasion and metastasis [12-15]. In fact, the ability of cucurbitacin E to inhibit filamentous actin (F-actin) depolymerisation led to the suggestion that it would be useful as a tool to study actin dynamics and actin-based processes in live cells [16].

With so many apparent biological activities, an important issue is how cucurbitacin compounds interact with and consequently inhibit their protein targets. This question is particularly important if a decision were made to use medicinal chemistry to optimize cucurbitacin compounds as anti-cancer therapeutics by improving their on-target selectivity and potency while minimizing their reported toxicities [17]. The mode of cucurbitacin binding to protein targets is also an important issue if they are to be used as chemical biology probes with confidence. One attempt to address this question used *in silico* docking of cucurbitacin B and E into the hydrophobic ligand-binding pocket of B-Raf [11]. However, no direct physical measurements were made to validate this hypothetical mechanism of action.

A previous attempt to characterize important cellular targets of cucurbitacin used biotinylated cucurbitacin E to purify interacting proteins from lysates of U937 human leukaemia cells and mass spectrometry (MS) for protein identification, which led to the discovery of Cofilin1 as a major interacting protein [12]. In this study, we aimed to examine how cucurbitacin analogues affect Cofilin1 activity and to identify the mechanism of action. Using Cofilin1 as an exemplar, we sought to determine if there might be specific or general modes used by cucurbitacin compounds to interact with target proteins, and by inference whether these compounds could potentially be optimized for potency and selectivity.

## Results and discussion

**Figure 1:**
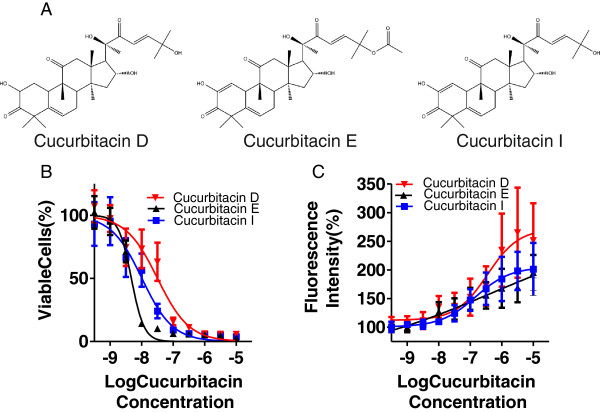
**Cytotoxicity of cucurbitacin compounds. A)** Chemical structures of cucurbitacin D, E and I. **B)** Cytotoxicity dose–response profiles for cucurbitacin D, E and I. Viable MCF7 breast cancer cell numbers were determined by counting DAPI stained nuclei 3 days after cucurbitacin treatment at doses from 0.3 nM to 10 μM with means and SEM depicted for 9 replicates. **C)** Relative single cell fluorescence intensity of phalloidin stained F-actin measured 4 h after cucurbitacin addition at doses from 0.3 nM to 10 μM. Means and SEM are depicted for 3 replicates.

To determine how cucurbitacins affect cell viability and the integrity of actin-based cytoskeletal structures, we tested the activities of cucurbitacin D, E and I (Figure 1A) on cell number and the fluorescence intensity of phalloidin-stained F-actin. MCF7 human breast cancer cells were treated with each cucurbitacin analogue at doses ranging from 0.3 nM to 10 μM for 3 days, then cell number was assessed by counting 4’,6-diamidino-2-phenylindole (DAPI) stained nuclei by high content imaging analysis (Figure 1B). The effect of each cucurbitacin was similar, with a rank order of potency of cucurbitacin E (EC_50_ = 4.7 nM) > cucurbitacin I (EC_50_ = 10 nM) > cucurbitacin D (EC_50_ = 32 nM). Interestingly, the −2.1 slope of the cucurbitacin E curve suggested more than one target for the cytotoxic effects. The gross effects of a range of cucurbitacin doses on the fluorescence intensity of phalloidin-stained F-actin was determined by measuring relative single cell phalloidin-staining 4 hours after compound treatment (Figure 1C). As with cell number, the effect of each cucurbitacin was similar, with a rank order of potency; cucurbitacin I (EC_50_ = 0.15 μM) > cucurbitacin D (EC_50_ = 0.35 μM) > cucurbitacin E (EC_50_ = 0.56 μM). The 0.2 slope of the cucurbitacin E curve again suggested more than one cellular target for effects on phalloidin staining of F-actin structures. These data indicate that cucurbitacins are more effective at reducing cell number than for inducing increased F-actin levels, suggesting that the cytotoxic properties of these compounds may be largely independent of their actions on cytoskeletal rearrangements. Evidence from cucurbitacin E in particular suggests that there may be multiple targets that decrease cell proliferation and influence phalloidin staining of F-actin.

**Figure 2:**
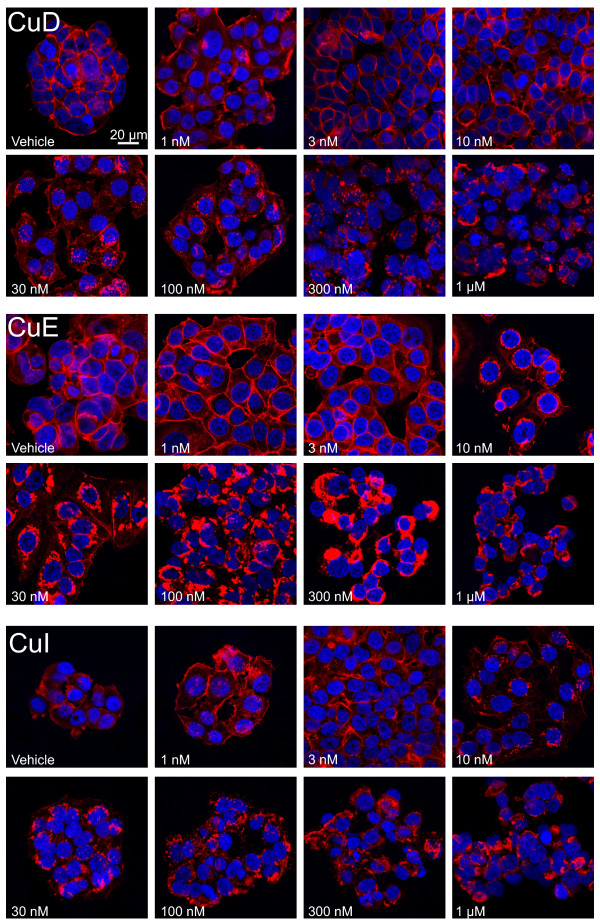
**Dose dependent effects of cucurbitacin D, E and I on the actin cytoskeletal structures in MCF7 breast cancer cells.** 4 hours after cucurbitacin D (CuD), cucurbitacin E (CuE) or cucurbitacin I (CuI) treatment at doses from 1 nM to 1 μM, cells were fixed, permeabilized and stained with Texas Red-conjugated phalloidin and DAPI. All images were acquired at identical magnification, but adjusted to produce images with equivalent phalloidin intensities for visualization of actin structures.

We next determined how cucurbitacin treatment qualitatively affected F-actin structures. MCF7 cells were treated with compound concentrations ranging from 1 nM to 1 μM for 4 hours, then after fixation, permeabilization and staining with DAPI and phalloidin, images acquired by confocal microscopy (Figure 2). The lowest concentration at which F-actin rearrangements were evident was 10 nM for cucurbitacin E and I, and 30 nM for cucurbitacin D. F-actin was progressively reduced at cell-cell interfaces and accumulated as large masses within the cytoplasm with increasing concentrations of each cucurbitacin. Although the fluorescence intensity of phalloidin stained F-actin was least potently affected by cucurbitacin E (Figure 1C), qualitatively it appeared to have the greatest effect on inducing rearrangements (Figure 2).

**Figure 3:**
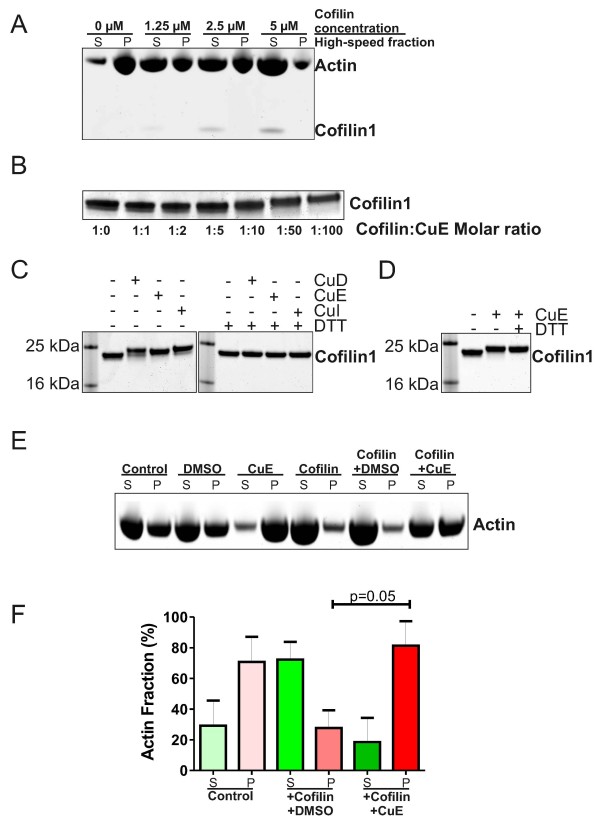
**Cucurbitacin compounds react with Cofilin1 and inhibit activity. A)** Ultracentrifugation was used to separate actin into soluble (S) monomeric actin and pellet (P) F-actin high speed fractions. Coomassie staining revealed that the addition of increasing Cofilin1 concentrations shifted actin from P to S fractions, indicating increased actin severing. The 5 μM Cofilin1 concentration was used for subsequent experiments. **B)** Cofilin1 at 5 μM was incubated with cucurbitacin E (CuE) at the indicated molar ratios. Protein mobility was slowed at 1:50 and 1:100 ratios. **C)** Incubation with cucurbitacin D (CuD), E or I (CuI) slowed Cofilin1 electrophoretic mobility, while co-incubation with 5 mM DTT blocked this effect on Cofilin1 mobility. **D)** Mobility shift of Cofilin1 could not be blocked if 5 mM DTT was added after incubation with cucurbitacin E. **E)** Actin partitioning into soluble (S) and pellet (P) fractions after ultracentrifugation was used to determine how actin-severing by Cofilin1 was affected by Cucurbitacin E. **F)** Quantification of the proportions of soluble (S) versus pelleted (P) actin fractions following ultracentrifugation for the indicated conditions. Cofilin1 increased the soluble monomeric actin fraction relative to control. Cucurbitacin E significantly (p = 0.05) inhibited Cofilin1-mediated actin severing relative to control DMSO vehicle when the P fractions were compared by Student’s t-test (n = 3).

The effect of cucurbitacin E on increasing cellular F-actin has recently been attributed to its direct conjugation with Cys257 on polymerized actin, but not monomeric globular actin (G-actin) [16]. An additional actin regulator identified as a potential target is the F-actin severing protein Cofilin1, which was isolated as a biotin-linked cucurbitacin E interacting protein [12]. To determine how cucurbitacin E might influence Cofilin1 F-actin severing activity, we first determined a Cofilin1 concentration that robustly reduced the level of F-actin that could be pelleted from 16.8 μM total actin by high speed ultracentrifugation. Actin was first polymerized *in vitro,* and then G-actin (supernatant fraction S) fractionated from F-actin (pelleted fraction P) by centrifugation at 150,000 *X* g for 1.5 hours (Figure 3A). Increasing Cofilin1 concentrations revealed an efficient effect of shifting actin from P to S fractions at 5 μM. To test the possibility that cucurbitacin E binds covalently to Cofilin1 as it does for actin [16], a range of cucurbitacin E (CuE) concentrations at molar ratios up to 1:100 (relative to constant 5 μM Cofilin1) were incubated with purified Cofilin1 protein for 16 h and examined for their ability to induce a Cofilin1 mobility shift on 12% Bis-Tris polyacrylamide gels. Although low cucurbitacin E concentrations had no obvious effect, reduced Cofilin1 mobility was clearly evident at 1:50 and 1:100 molar ratios (Figure 3B). Covalent modification of actin was similarly achieved with a 1:100 molar ratio of actin protein to cucurbitacin E [16]. To determine whether similar effects were seen with additional cucurbitacin compounds, each of three cucurbitacin compounds was incubated at 1:100 molar ratio with Cofilin1 that had been dialysed to remove dithiothreitol (DTT). The mobility of Cofilin1 on 12% Bis-Tris polyacrylamide gels was slowed by each cucurbitacin (Figure 3C, left panel), consistent with stable modification of Cofilin1 by cucurbitacin compounds and increased mass. The inclusion of 5 mM DTT blocked the mobility shift induced by cucurbitacin compounds (Figure 3C, right panel). However, there was no effect on the Cofilin1 mobility shift if DTT was added after the incubation of cucurbitacin E with Cofilin1 (Figure 3D). These results indicate that Cofilin1 was modified by cucurbitacin compounds to produce stable modifications that could not be reversed by DTT.

The actin severing assay was used to determine whether cucurbitacin E inhibited Cofilin1 F-actin severing activity (Figure 3E). While DMSO vehicle control did not change the S/P ratio, cucurbitacin E notably increased the pelleted F-actin, consistent with a direct effect on inhibiting depolymerisation [16]. The effect of Cofilin1 was to shift actin to the monomeric S fraction, which was unaffected by DMSO vehicle control. In contrast, treatment with cucurbitacin E inhibited the actin severing activity of Cofilin1, since the P fraction was significantly (p = 0.05) increased relative to DMSO-treated Cofilin1 samples (Figure 3F).

**Figure 4:**
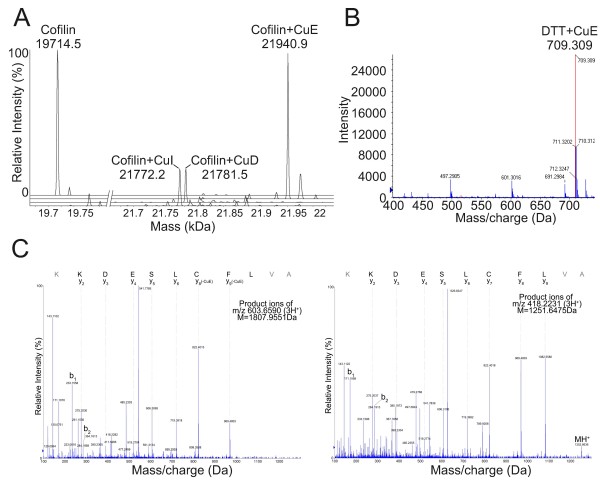
**Mass spectrometry reveals conjugation of cucurbitacin to thiols. A)** Mass of Cofilin1 protein alone or following incubation with cucurbitacin D, E or I as indicated, as determined by mass spectrometry. **B)** Mass/charge determination of cucurbitacin E that had been incubated with DTT reveals that a reaction product that conserves mass had been formed, consistent with a Michaels addition reaction. **C)** MS-MS spectra of the tryptic peptide containing Cys39, modified (left) and unmodified (right) with cucurbitacin E. Correspondent mass of the precursors peptides are indicated in the figures, and highlight that the same peptide sequence has a difference in molecular weight, corresponding to one cucurbitacin E molecule (556.3 Da).

Mass spectrometry revealed that each cucurbitacin treated sample had an increased mass (Figure 4A) corresponding to the mass determined for Cofilin1 (19,714.5 Da) plus four times the mass of each cucurbitacin compound used (cucurbitacin D hypothetical/measured masses = 21781.3/21781.5; cucurbitacin E = 21941.3/21940.9; cucurbitacin I = 21772.9/21772.2). Incubation with 10 mM DTT for 3 h resulted in virtually complete reaction of 100 μM cucurbitacin E with no loss of mass in the product of the two reactants (hypothetical/measured mass/charge = 709.308/709.309) (Figure 4B). To determine the exact binding sites of cucurbitacin E, the treated Cofilin1 was digested with trypsin, and the fragment sizes determined by MS. Fragmentation by MS-MS of the peptide containing Cys39 revealed that this was a site of cucurbitacin E conjugation (Figure 4C). Fragments corresponding to cucurbitacin E binding to peptides containing cysteines 80 and 139 were also clearly identified, with weaker evidence for binding of cucurbitacin E to cysteine 147 (data not shown).

**Figure 5:**
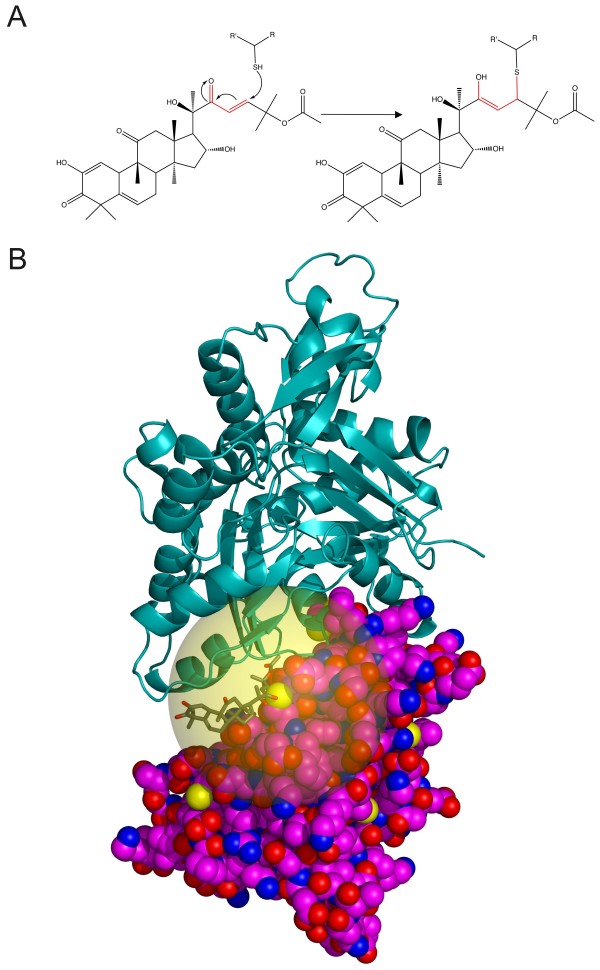
**Model of cucurbitacin binding to Cofilin1. A)** Reaction of cucurbitacin E (Michaels acceptor group in red) with nucleophilic thiol, producing a product that conserves mass. **B)** Crystal structure of human Cofilin1 (space filled) modelled with associated actin (ribbon), based on the C-terminal Cofilin-like domain of mouse twinfilin (Twf-C) in complex with actin. Cucurbitacin E (sticks) is modelled as conjugated to Cys139, with its potential rotational space illustrated by the transparent yellow sphere. The size of cucurbitacin E at this position would likely clash with actin, resulting in reduced actin-severing activity.

As there was no loss of mass when cucurbitacin compounds bound to Cofilin1 protein (Figure 4A) or DTT reacted with cucurbitacin E (Figure 4B), it is likely that the covalent binding is in the form of a thioether bond, formed by the reactive α,β keto group on the cucurbitacin undergoing a Michaels addition to Cofilin1 cysteine thiols (Figure 5A).

It is worth noting that the cucurbitacin-conjugated Cofilin1 became extremely hydrophobic with a marked propensity for non-specific binding to dialysis membranes and columns, which resulted in complete loss of detectable cucurbitacin-conjugated Cofilin1 in some procedures. Only by treating proteins directly in solution or following extraction from polyacrylamide gels could any material be obtained for analysis. Although Cofilin1 was identified as a biotinylated-cucurbitacin E interacting protein in cell lysates by mass spectrometry [12], no mass shift was detected after incubation of purified Cofilin1 or Gelsolin with cucurbitacin I [18], likely due to the loss of cucurbitacin-conjugated protein because of their hydrophobicity. This property has also likely hampered efforts to identify additional cucurbitacin binding proteins.

**Figure 6:**
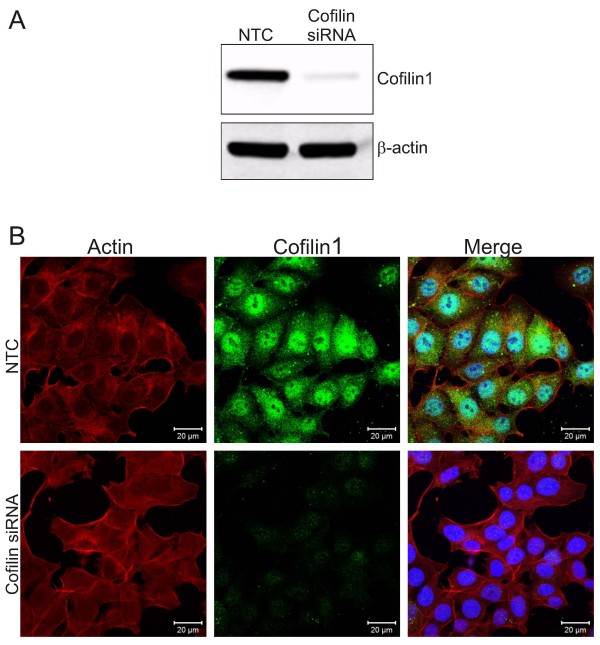
**Cofilin1 knockdown does not affect F-actin structures. A)** Western blotting of MCF7 lysates indicating efficient siRNA-mediated Cofilin1 knockdown but no effect of non-targeting control (NTC) siRNA. **B)** Immunofluorescence of F-actin and Cofilin1 following transfection with NTC or Cofilin1 siRNA revealed that Cofilin1 inhibition is not sufficient to induce significant F-actin changes.

To determine whether Cofilin1 inhibition would be sufficient to induce the effects on F-actin structures observed following cucurbitacin treatment (Figure 2), we used siRNA to knockdown Cofilin1. Western blotting showed effective suppression of Cofilin1 protein in MCF7 cells transfected with Cofilin1 siRNA but not non-targeting control (NTC) siRNA (Figure 6A). Staining transfected cells revealed that Cofilin1 knockdown was not sufficient to reproduce the effects on F-actin structures induced by cucurbitacins (Figure 6B), consistent with the conclusion that there are multiple proteins targets for these compounds.

**Figure 7:**
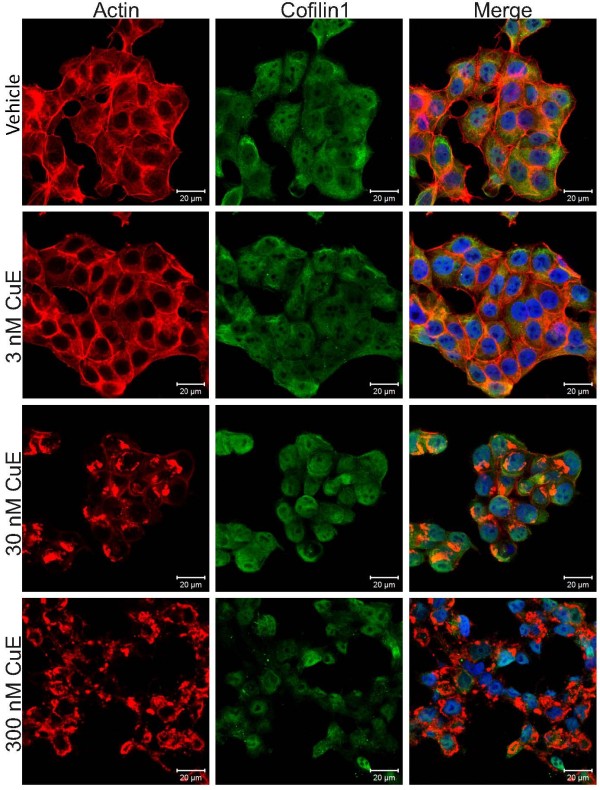
**Subcellular localization of Cofilin1.** Increasing concentrations of cucurbitacin E (CuE) from 3 nM to 300 nM were incubated with MCF7 cells for 4 h, followed by fixation and staining for F-actin structures and Cofilin1 subcellular localization. Despite evident effects on F-actin and cell morphology at 30 nM and 300 nM cucurbitacin E, Cofilin1 distribution was not observably changed.

The sub-cellular localization of Cofilin1 was examined in MCF7 cells treated with DMSO vehicle or cucurbitacin E at 3 nM, 30 nM or 300 nM for 4 h. Fixed cells were stained for F-actin with phalloidin or Cofilin1 using the antibody validated in Figure 6. Although F-actin was found in large perinuclear masses at 30 nM and 300 nM cucurbitacin E, Cofilin1 distribution did not vary greatly from its mixed cytoplasmic/nuclear distribution (Figure 7).

Our results clearly show that cucurbitacin compounds bind covalently to the cysteines present in Cofilin1, and that this binding inhibits the ability of Cofilin1 to sever F-actin. By modelling the addition of cucurbitacin E to Cys139 on our recently solved crystal structure of human Cofilin1 (depicted with space filling; PDB ID 4BEX [19]) that was modelled with associated actin (ribbon) based on the C-terminal Cofilin-like domain of mouse twinfilin (Twf-C) in complex with a single unit of actin (PDB ID 3DAW) [20], it becomes apparent that the conjugated cucurbitacin would disrupt the interface between Cofilin1 and actin (Figure 5B). A similar conjugation to Cys147 would also likely lead to a clash with F-actin that would inhibit actin-severing. The increased hydrophobicity of the surface of cucurbitacin-modified Cofilin1 would also likely interfere with binding to F-actin. Although Cys39 and Cys80 are positioned away from the Cofilin1-actin interface, their relatively buried positions could result in disruption of protein folding if conjugated to the bulky cucurbitacin molecules, similar to the cucurbitacin-induced structural rearrangements observed for human serum albumin [21], which could also inhibit F-actin severing activity.

In addition to cucurbitacin E binding to thiols on F-actin [16], Cofilin1 and DTT, cucurbitacin B was reported to form adducts with thiol-containing N-acetylcysteine (NAC) and glutathione, but not thiol-free vitamin C or ascorbic acid [22]. Although NAC blocked the cytotoxicity of cucurbitacin B, vitamin C and ascorbic acid did not [22]. These results suggest that rather than inhibiting the effect of cucurbitacin B through anti-oxidant properties, which NAC, vitamin C and ascorbic acid all share, the inhibitory effect of NAC is more likely to be a consequence of its direct conjugation with cucurbitacin B. In fact, the ability of the reactive oxygen scavenging NAC to reverse the effects of cucurbitacin has widely been interpreted as evidence that generation of reactive oxygen species is part of its mechanism of action [23]. Our alternative interpretation is that the neutralization of the biological effects of cucurbitacin compounds is via direct conjugation with NAC in tissue culture medium or in cells, meaning that all experiments in which NAC and cucurbitacins have been combined should be interpreted with caution.

## Conclusions

Although cucurbitacin compounds have been proposed to be potential anti-cancer drugs and are used to inhibit specific signal transduction pathways, the results of this study and others [16,23] support the conclusion that cucurbitacins non-specifically bind protein targets by forming thioether bonds via a Michaels-type addition. This would allow cucurbitacins to be conjugated with a broad array of potential protein targets, many of which would be inhibited or disrupted as a consequence. As a result, their value as chemical biology probes is limited and must be confirmed by independent means. For example, although cucurbitacin I was reported to be a selective inhibitor of Jak/STAT3 signalling [6] and has been used to test the involvement of this pathway in various processes, the ability of cucurbitacin I to activate Rac1 was not replicated by Jak2 or Stat3 knockdown by siRNA [24]. The findings in this study also indicate that the binding mode of cucurbitacin compounds to protein targets means that optimization for selectivity would be unlikely to work, which would make it very difficult to minimize toxicities or improve the therapeutic window for future clinical development. Optimistically, alternative ways that their potential therapeutic utility could be improved in the future would be through targeted delivery to tumour cells, for example through antibody-conjugation or incorporation in liposome microparticles [25].

## Methods

### Cell culture

MCF-7 human breast cancer cells were grown in Dulbecco’s modified eagle’s medium (Gibco) with 10% fetal bovine serum (PAA) at 37°C and 5% CO_2_. Transfections with non-targeting control siRNA D001810-01-05 and human Cofilin1 siRNA J012707-05 (Abgene) were performed as described in [26].

### Cytotoxicity and actin-phalloidin fluorescence assays

MCF-7 cells were trypsinized and to each well of Greiner black-walled, clear bottomed 96-well plates, 100 μl of cell suspension (2,000 cells for cytotoxicity, 5,000 cells for phalloidin staining) was added. Plates were left to stand in the hood for 10 minutes to aid uniform cell attachment prior to placing in CO_2_ incubator. After 24 hours attachment, 100 μl of media containing DMSO vehicle or cucurbitacin compounds was added to the wells. For cytotoxicity, cells were cultured for a further 3 days and then after fixation with 4% paraformaldehyde for 15 minutes at room temperature and DAPI staining of cell nuclei, cell number was assessed using an Operetta high content imaging system (Perkin Elmer). For actin-phalloidin fluorescence measurements, cells were cultured for 4 hours after treatment. After fixation with 4% paraformaldehyde for 15 minutes at room temperature, permeabilization with 0.25% (v/v) Triton X-100 and staining of actin structures with Texas Red-conjugated Phalloidin (2 μg/mL), fluorescence intensity was measured using an Operetta high content imaging system. Imaging of actin structures was done using a Zeiss 710 confocal microscope.

### Binding of cucurbitacin to Cofilin1

Cofilin1 was expressed and purified as described previously [27]. Binding of cucurbitacin to Cofilin1 was achieved by incubating 5 μM Cofilin1 with a dose range from 5 μM to 500 μM cucurbitacin (solubilised in 100% DMSO) or an equivalent volume of DMSO at 4°C overnight. Cucurbitacin binding to Cofilin1 was analysed on 12% Bis-Tris SDS-PAGE gels and SimplyBlue (Invitrogen) stained gels scanned on a LiCor Odyssey.

### Mass-spectroscopy analysis

5 μmol Cofilin1 was incubated with 0.5 mM Cucurbitacin E, separated by SDS-PAGE, stained with colloidal Coomassie Blue and digested with trypsin. The digests were analysed by LC-MS using an AB-Sciex 5600 TripleTOF mass spectrometer coupled to an Eksigent 2D Ultra HPLC system fitted with a 150 × 0.075 mm C_18_ packed emitter (New Objective). Digests were loaded in 2% acetonitrile/0.1% formic acid (Buffer A) and then eluted with a linear gradient of buffer B (100% acetonitrile/0.1% formic acid) at 300 nL/min. LC-MS data was searched using Mascot 2.4 run on a local server against SwissProt allowing for trypsin cleavages and cysteine modifications of oxidation and cucurbitacin isoforms. To determine the intact mass of cucurbitacin modified Cofilin1, complexes were diluted into 25% acetonitrile/0.5% formic acid and separated on an Eskigent 150 × 0.3 mm ChromXP C18CL column. The mass spectra were acquired on the 5600 TripleTOF with the intact protein script activated (AB-Sciex) from m/z 600–2000 and spectra were deconvoluted using BioAnalyst 1.5 to calculate the exact mass of the protein complex.

100 μM Cucurbitacin E was incubated plus or minus 10 mM DTT in 50 mM TEAB pH 8 for 3 h at room temperature and diluted 1:10 with 50% acetonitrile/0.1% formic acid. Samples were analysed by MS on 5600 TripleTOF by infusing at 1 μl/min and data was acquired in negative ion mode (TOF-MS and MS-MS).

### F-actin depolymerization assay

The Actin Binding Protein Biochem Kit with Non-Muscle Actin (Cytoskeleton) was used according to manufacturer’s instructions. Aliquots of actin polymerization buffer and ATP were rapidly defrosted in a room temperature water bath and kept on ice. Cofilin1 samples were centrifuged for 1 h at 100,000 *X* g at 4°C, supernatants taken and kept on ice as test protein stocks. General actin buffer was supplemented with 0.2 mM final ATP. One 250 μg aliquot of actin was diluted to 1 mg/ml with 225 μl of ATP-supplemented general actin buffer, mixed to ensure complete re-suspension and left on ice for 30 min. 25 μl of actin polymerization buffer was pipetted into the actin protein and mixed well. The actin was incubated at room temperature for 1 h and kept as F-actin stock at 21 μM actin. All tubes were incubated at room temperature for 30 min. Afterwards the tubes were centrifuged at 150,000 *X* g for 1.5 h at 24°C. Supernatants were removed and kept on ice. To each tube 10 μl of 5× Laemmli reducing sample buffer was added. The pellets were re-suspended in 30 μl water by mixing for 2 min, leaving on ice for 10 min and repeated mixing. The samples were transferred into tubes and supplemented with 30 μl 2× Laemmli reducing-sample buffer. The samples were frozen at −20°C until further analysis by SDS-PAGE. Quantification was performed with a Licor Odyssey by scanning the fluorescence intensity of SimplyBlue (Invitrogen) stained bands at 700 nm.

## Abbreviations

DAPI: 4’,6-diamidino-2-phenylindole; DTT: Dithiothreitol; EC50: Half maximal effective concentration; F-actin: Filamentous actin; G-actin: Monomeric globular actinMS mass spectrometry; SDS-PAGE: Sodium dodecyl sulphate polyacrylamide gel electrophoresis; TOF: Time of flight; Twf-C: C-terminal Cofilin-like domain of mouse twinfilin.

## Competing interests

The authors declare that they have no competing interests.

## Authors’ contributions

MG, MS, JM, DB, JC, MB, AM, SL and NM performed experiments, MG, MB, SL, NM and MFO analysed and interpreted the data, MG, MS, JM, SL and MFL prepared the figures. MFO designed the research, MG and MFO wrote the manuscript. All authors had a final approval of the manuscript.
